# Contested identities in Europe: Historical insights into the construction of citizenship education from the bottom up

**DOI:** 10.1177/14749041251403832

**Published:** 2025-12-17

**Authors:** Anja Giudici, Thomas Ruoss, Susannah Wright

**Affiliations:** Cardiff University, UK; Swiss Federal University for Vocational Education and Training, Switzerland; Oxford Brookes University, UK

**Keywords:** Citizenship education, Europe, history of education, education policy

## Abstract

Citizens need to be educated. However, both understandings of citizenship and the education required to disseminate them are inherently contested. After presenting an analytical framework that allows for a conceptually grounded analysis of such contestation, this introduction draws on the papers included in the special issue to identify three axes that have structured the debate around citizenship education in modern Europe: the roles of the state, of place, and of conflict versus consensus. In conclusion, we present the papers included in this special issue and consider their value in both showcasing the diversity of understandings of citizenship in contemporary Europe, as well as the issues that this diversity raises for education research.

## Contested education and contested citizenship

‘Nothing could be more crucial to democracy than the education of its citizens’ Nussbaum (2006: 387) famously stated. A long tradition of educational and political theorists has argued that any discussion of citizenship norms and practices must also consider the institutions that produce them – including, first and foremost, educational institutions (Biesta, 2010; Callan, 2004; Gutmann, 1999; Wahlström, 2022). Indeed, abundant empirical literature shows that education plays a crucial role in forming citizenship behaviour and norms (Galston, 2001; Geboers et al., 2013). But what kind of citizens should educational institutions shape?

Answers to this question are inherently contested. The emergence and institutionalisation of modern education systems in 19th- and early 20th-century Europe provided reformers with a powerful tool to shape future citizenries (Harp, 1998). The understandings of citizenship of then dominant forces, however, differed widely. Repeated, sometimes violent, struggles erupted over whether schools should forge religious or secular; stratified or equal; national, regional, or global; pacifist or militarily equipped citizenries. Churches and social reformers, cultural majorities and minorities, as well as liberal, conservative, democratic, socialist, authoritarian, and monarchist parties all held different understandings of citizenship, and therefore fought for different forms of citizenship education (Brubaker, 2004; Tröhler et al., 2011).

Compared to this historical legacy, the debate around citizenship education experienced by recent generations seems almost consensual. World culture scholars relate this development to the growing influence of international and multi-national organisations and networks. From the League of Nations’ educational bodies, through UNESCO, and including the European Union, such organisations have defined and disseminated common citizenship norms steeped in what Schofer et al. (2022: 510) call the ‘liberal international order’. From the perspective of world culture theory, a global and consensual notion of citizenship has therefore come to shape national understandings of citizenship, and related educational policies (Furuta, 2020; Lerch et al., 2017). Familiar statements about citizenship education developing ‘active, informed and responsible citizens’ who ‘interact effectively, think critically, act in a socially responsible way and democratically’ (European Commission, 2017: 9) indeed suggest there is a neutral and common understanding of the kind of individuals and societies education should aim for.

Recent developments question the existence of such a neutral and common understanding. Across Europe, innovations in citizenship education have been accompanied, and transformed, by public debate and contest. The 2014 governmental requirement for English teachers to convey ‘British values’ in their classrooms triggered a wave of discussions, not only on how this should be achieved, but on the very meaning of ‘Britishness’ (Vincent and Hunter-Henin, 2018). More recently, the far-right party Alternative für Deutschland called for the closure of the state-run centre for political education in the state of Saxony-Anhalt, arguing that it was indoctrinating students, and should be replaced by an institute for civic education and cultural identity (Der Spiegel, 2025). Curricula promoting inclusivity and sustainable development are increasingly under attack across Europe and have been overturned in some states, such as Hungary (Neumann, 2023), and, across the Atlantic, in the United States (The White House, 2025). Schofer et al. (2022: 510) place such developments in the context of the broader decline of global liberal norms over the past few decades. This decline, they argue has created space for and ‘invigorated alternative cultural frameworks’.

Against the backdrop of such contestation, how is citizenship education developed, and by whom? The articles included in this special issue highlight the wide range of understandings and declinations of citizenship norms that have characterised recent European history, as well as the diverse range of actors that have developed and disseminated them. Spanning the last century and the European continent, the articles empirically substantiate a core proposition of education and political theory, namely that understandings of education are constituted by ideals about what a community ought to be – and that both citizenship norms and the outlook of the institutions meant to produce them are therefore inherently controversial (Biesta, 2010; Gutmann, 1999). Whether acting primarily through education or politics, or across a mix of both, the actors analysed in these pages relate their different understandings of citizenship to different versions of citizenship education.

This special issue focuses on specific groups’ understandings of citizenship, how they relate these understandings to education, and how they have worked to implement them. Therefore, the articles adopt an historical focus that considers educational systems in their wider political, cultural, and ideological contexts. They constitute a carefully selected ensemble of historical case studies of political and educational actors from different European contexts and confront these actors with a common set of questions. What aspects of dominant understandings of citizenship did these actors contest and what counter-ideals did they propose? Which conceptions of citizenship education complemented these ideas? How did these actors seek to introduce their conceptions of citizenship education into the educational debate and implement them in practice?

By answering these questions historically, the articles provide significant empirical contributions to our understanding of the heterogeneous ideals of citizenship that have circulated in modern Europe, and the importance that those who hold these ideals attribute to education in forming citizens. They specifically focus on the ideals and strategies of often overlooked movements and actors. They contribute towards an understanding of citizenship education that is aware of frictions and controversies as well as consensus, and which integrates inputs of groups that act at least partly outside state and supra-national institutions. At the same time, the analysis of contested understandings of citizenship education as championed by multiple and diverse groups can be understood as a practice through which the nation as a political and cultural phenomenon can emerge in the first place (Piattoeva et al., 2023).

Together, the articles in this special issue also constitute the groundwork for re-thinking the concepts and theories underlying research on citizenship education. The next section of this introduction sketches a common framework that allows the specific cases represented in the articles to speak to each other. It also discusses implications for research on citizenship education in Europe, including its theory, politics, history, and practice.

## Framework

Citizenship is a concept with a long history and multiple meanings. This special issue is concerned with specific understandings of citizenship as put forward by collective actors, rather than with the behaviours or values of actual citizens. Political scientists label such understandings ‘citizenship norms’, defined as ‘a set of expectations about the citizen’s role in politics’ (Dalton, 2008: 78) or, more broadly, ‘the ideals and virtues that citizens should develop [. . .] in order to secure the justice and stability of the polity to which they belong’ (Callan, 2004: 73).

We draw on, but expand, this definition of citizenship norms. In keeping with Biesta (2010) and Lister (2003) we argue that citizenship norms typically reach beyond politics proper and into other social spheres such as the economy or family. Indeed, the Active Citizenship Composite Indicator – the leading European measurement of citizenship behaviour – asks respondents about their engagement in politics, community life, and civil society (Hoskins et al., 2006; see also Sandoval-Hérnandez et al., 2018). The boundaries between such spheres are highly contested. We therefore understand citizenship norms as expectations about citizens’ role in society, and about the related values and skills they should develop. Adopting a historical perspective, we use this broad definition to interrogate actors’ own norms and definitions, to understand which social boundaries they draw, and which spheres they prioritise.

Citizens are not born with citizenship-related values and skills. They develop them in educational and other settings. Formal education, with its systematic and institutionalised nature, provides a powerful tool for those in power to purposively ‘stimulate the growth of citizens in the making’ (Marshall, 1965: 81). Different experiences can influence the direction of this growth. The content someone receives during their schooling, how they are taught to interact with friends and authorities, which peers they encounter as a result of school selection and choice, their say in school matters, and so much more, contribute to shaping how they will engage with politics and society as citizens. In this sense, ‘all education is civic education’ (Galston, 2001: 219). The scope of this special issue therefore goes beyond the specific subject of citizenship or civic education. We define citizenship education as any educational institutions and practices aimed at fostering specific citizenship norms – regardless of whether this involves designing curricula, re-structuring institutions and certificates, or developing spaces and infrastructures.

The relationship between education and citizenship norms is a core question in educational and political philosophy (Callan, 2004; Galston, 2001; Gutmann, 1999). This literature describes this relationship as inherently contested. Different views on what constitutes a just regime and society typically entail disagreement on citizenship norms. But a common social vision does not solve the issue either. Agreement on what constitutes a just regime and society can still hide disagreements about the values and skills citizens ideally need to uphold this regime – as shown by recurrent debates on what being a citizen in a liberal democracy implies (Galston, 2001; Gutmann, 1999). From an educational perspective, moreover, agreement about such values and skills can still come with further disagreement on the educational institutions and practices needed to convey them (Biesta, 2010).

We believe historical inquiry to be particularly well equipped to explore what lies behind seemingly unified rhetorics on citizenship education. Our approach focuses on collective actors, defined as groups pursuing one or more common goals through joint, intentional action (Scharpf, 2006). Rather than following actors representing European institutions (e.g. European Commission, 2017), we consider actors *within* Europe in order to strengthen an exploratory, historiographical perspective on the development of contested ideals of citizenship education. From an analysis of the structure and practice of contestation over citizenship education in diverse historical and geographical settings we derive insights into the role of political and social views in shaping educational views and, vice-versa, the role attributed to schooling as means to reach actors’ ideals of society.

This special issue does not adopt a comparative approach. The contributions offer a contextualised analysis of the ideas and strategies of specific actors which ‘speak’ to one another by exploring the dynamics outlined below. They examine how these actors navigate their specific political and educational landscapes and dominant citizenship norms, as well as the mechanisms through which they themselves contribute to shaping such norms in particular contexts. This type of analysis enables us to draw overarching findings based on parallels in the structure of debates and practices in different contexts, while also providing insights into the specific dynamics of a selection of intriguing cases.

## Overarching findings: Axes structuring debates and practices

With this special issue we aim to showcase the value of historical research in revealing the nature and influence of different views of citizenship education. The papers harness the strength of historical research to explore the complex and dynamic relationship between social and political ideals and education in particular contexts and considering a diverse set of actors. Still, together, they pinpoint three conceptual axes. These axes are not specific to each case; rather, they appear across cases, shaping the debates around citizenship and how actors position themselves and their strategies within these debates across time and places. We developed these axes through an iterative process orchestrated through several ECER symposia within Network 17 Histories of Education (Geneva in 2021, Nicosia in 2022, and Glasgow in 2023), as well as a workshop funded by the Swiss National Science Foundation (Zollikofen/Bern in 2023). Together, they result in a framework that allows the papers to speak to one another whilst identifying structuring elements that are of central importance for the analysis of understandings of citizenship from historical and other educational perspectives. We discuss each axis separately below, relating the insights from the special issue to existing research in education, history, and politics.

### Citizenship education and the state

Most empirical studies of citizenship education focus on norms related to states. They typically examine the representation of these norms in policy, and in official curricula (Giudici, 2023). They emphasise the extent to which state authorities have relied on formal education to promote their vision of citizenship. For modern states, Gellner (1983, 34) famously argued, the ‘monopoly of legitimate education is now more important, more central than is the monopoly of legitimate violence’.

However, states are hardly unitary actors. They are complex organisations comprising actors with different logics, interests, and views (Binder, 2009) – including views relating to the boundaries and identity of the nation itself (Brubaker, 2004). At the same time, while official (education) policy is within the remit of state authorities, it is often shaped by groups that operate outside of, and sometimes in opposition to, state institutions (Giudici, 2023; Niesz et al., 2018; Wright, 2017).

The studies in this special issue add to existing work (Englund, 1996; Hay, 2014; Rockwell and Roldán Vera, 2013), including work recently published in this journal (Wahlström, 2022), in advocating the benefits of a pluralistic and relational understanding of the state and its politics. At the same time, they, first, highlight the range of actors such an understanding requires us to consider. When it comes to citizenship education, actors as diverse as political extremists, missionaries, savings bankers, and teacher trainers have, among others, engaged with state authorities to have their views inscribed in official policy. Secondly, the studies show that states – even authoritarian ones – are neither insulated nor unitary ‘actors’. State structures include groups and parties whose experiences and views on the relationship between citizenship and education can differ greatly. Finally, the articles demonstrate that social and educational reformers within and outside state structures do not operate in isolation, but rather develop and refine their strategies and norms in relation to prevailing citizenship norms and educational contexts. Therefore, recognising and analysing the relationship between state authorities and the actors involved in reshaping citizenship through education represents an important first axis of analysis of citizenship education and its contestations.

### Citizenship education and place (local to global)

The focus of legal citizenship status, and therefore of formal citizenship education, has traditionally been on the nation, typically the nation state as the space defined by (sometimes contested) international borders. The nation state, however, is just one unit in the ‘social-moral-political strata of human groupings’ (Heater, 2004: 194). Individuals might experience strong ‘social and ethical connections’ (ibid.) to groupings above the nation state – international ones – and below the nation state – regional or local ones. Multiple citizenships at the same time are possible. The actors in this volume have targeted their efforts at groupings at the local, national, and international levels through their educational schemes.

Within the ‘multi-scalar geographies’ (Mills, 2022: 2) of citizenship education, place operates in complex ways. International influences can inform nationally or locally-based actors, and local and national influences can inform internationally-based actors. International organisations can operate through local groups. Projects led by organisations at a local or national level can have internationally-focussed aims, while international organisations can coordinate educational projects with locally-focussed aims. The ‘imaginative and institutional geographies’ (Mills and Waite, 2017: 66) of education for citizenship are not necessarily identical. They can coalesce or pull in different directions. Actors can see the local, national, and international as complementary, contentious, or sometimes both at the same time, in terms of their aims and processes. Place within citizenship education, then, introduces the potential for contestation and tension between actors, and also introduces ambiguity for individual actors in what they were trying to achieve and their strategies for achieving it.

### Citizenship education, conflict and consensus

Education and political philosophers have long criticised the idea that the norms underlying citizenship education are, or can be, neutral and consensual (Biesta, 2010; Joris, 2022). Media reports and historical studies often shine the spotlight on particularly visible episodes of contestation. For example, when parents or social movements take to the streets to demand schools align with more progressive or traditional visions of society, or to oppose school integration (Mason, 2009; Niesz et al., 2018).

By following specific actors rather than episodes of contention, the articles in this special issue show that contestation does not have to be openly visible to exist. Groups advancing different visions of citizenship education may not act in public, or they may strategically play down their differences. They may be considered illegitimate, unworthy of historical record, or they may act in authoritarian contexts where expressing opposition is dangerous. In their theory of resistance, Hollander and Einwohner (2004) distinguish between ‘overt’ and ‘covert’ resistance. The latter is only effective if it is not recognised as resistance by those in positions of power at that moment. Contestation in the articles included in the special issue can also be partial, with aims, actions and rhetoric of the actors examined sometimes combining with and sometimes opposing those of other contemporary actors articulating views on citizenship education.

These three axes represent common structuring elements that could not have been developed without prior methodologically rigorous and theoretically informed historical analysis. The papers in this special issue all employ meticulous empirical analysis, making use of a variety of primary archival sources authored by both individuals and collectives, including pedagogical materials, educational pamphlets, political manifestos, oral histories, official government reports, as well as internal and public-facing communication from political and economic organisations. They use these sources creatively to analyse different aspects of actors’ understanding of citizenship education, as well as the strategies actors use to realise these understandings politically and pedagogically – mobilising frameworks ranging from postcolonial and electoral to emotion-related theories. These articles not only add to our theoretical and conceptual knowledge, but also provide fascinating insights into the variety of citizenship norms that have existed in Europe, and how these norms have been related to and realised in education.

## Specific findings: An overview of the papers

Together, the eight papers included in this special issue focus on the educational views and political strategies of a wide range of movements, contributing towards a history of 20th century European citizenship education from the bottom up. All of the papers address the three aforementioned axes, albeit in different ways. We present them in chronological order, moving from more state-focussed studies to analyses of actors interacting with state institutions from outside.

The first study, by Buchardt (2025), transports us to Denmark in the 1880s–1930s. Focussing on publications by the ‘meso-level’ actors involved in both defining and disseminating understandings of citizenship through education, Buchardt compellingly demonstrates that such understandings, while tied to the needs and ideals of state-building, are never monolithic. Instead, they are forged by the imagined or actual experiences of these particular actors, including public intellectuals inspired by lessons they drew from colonial government. Buchardt’s analysis shows that conceptions of citizenship themselves are not monolithic either but can be carefully designed to promote cohesion while preserving hierarchies and difference based on characteristics such as class or race.

The second paper, by Van Ruyskensvelde et al. (2025), analyses the role of citizenship education in the promotion of national consciousness in Belgium, with a focus on the aftermaths of the First and Second World Wars. The authors show that during these periods discussions about citizenship education were shaped by sometimes conflicting allegiances to national, regional, and international patriotism and solidarity. Drawing on archival materials from selected teacher training colleges, the authors emphasise the role of teacher training curricula as a site of contestation over citizenship and how to educate those responsible for the moral and civic formation of future generations.

Darvai and Somogyvári (2025) explore discussions around citizenship education in Hungary during the socialist Kádár Era (1957–1985). Their extensive archival analysis allows us to glimpse behind the curtain of a non-democratic system. It reveals that those officially responsible for education, including ministers, professors, and teachers, held different understandings of citizenship education. These differences resulted in contradictory and continuously renegotiated official policy, despite an apparent unitary political ideology and a lack of democratic institutions. Darvai and Somogyvári therefore emphasise the importance of considering hidden forms of resistance and contestation, as well as of carefully reviewing whose views are actually inscribed in and disseminated through state education.

With the fourth study, our focus shifts from diverse actors operating within state institutions to non-state actors interacting with state authorities. In their article, Groves and Navarrete-Sánchez (2025) take a close look at the ideas and actions of a leading Catholic parents’ association in democratic Spain between 1978 and 2006. Their analysis shows that, rather than passively accepting the secularisation inscribed in state institutions following the democratic transition, the association strove to preserve religious understandings of citizenship education and the institutions and practices that disseminated them. Groves and Navarrete-Sánchez trace how the association adapted its religious understanding of citizenship education and the strategies employed to defend it over an extended time-period, weathering political change in order to maintain its influence.

Wright (2025) attends to the role of emotions in connexion with conceptions of world citizenship among young people promoted by the League of Nations Union, a voluntary association, in Britain (1919–1939). By examining texts published by the League of Nations Union and by young people, she demonstrates the multi-scalar nature of debates on citizenship education and how these debates can connect the intimate and local to the global. Her analysis also reveals the importance of not limiting investigations of citizenship education to (academic) knowledge. It reveals the emotional terrain of hope, fear, and international friendship envisaged by the League of Nations Union and articulated and experienced by young people themselves.

Ruoss (2025) analyses the activities of the International Thrift Institute, a globally active private interest group in the banking sector, from the 1920s to the 1960s. His analysis powerfully demonstrates that groups from outside the partisan-ideological sphere and state institutions can also develop and advocate new approaches to citizenship education. Operating across diverse political systems, this group had to present a consensus on the type of economic citizen they wanted to champion, but this consensus was forged on, and often reflected, conflicting views and political requirements.

Giudici and Pultar (2025) focus on another political actor that, until recently, operated outside state institutions: the post-1945 European far right. Their article shows that, despite their characteristic illiberal or anti-democratic attitudes, far-right groups have championed different types of citizenship norms and related educational policies. By comparing and contrasting the norms and policies championed by different ideological strands and types of actors within the movement, they show that these differences are both ideological and strategic, raising questions about the role of partisan and electoral considerations in shaping educational ideals.

In her *synthesising paper*, Margot Joris offers an institutional European perspective on citizenship education that complements the focus on particular actors and states in the other papers within this special issue. Joris examines citizenship education through a ‘tectonic’ lens, questioning both its ability to solve currently topical crises and the idea that more education automatically produces better citizens. Her contribution does not only further theorises the inherently normative, contested, and unpredictable nature of citizenship, but also highlights the normative desirability to maintain an open understanding of citizenship that leaves space for generational renewal, democratic discussion, and pedagogical autonomy.

We are proud of this special issue, which we believe provides compelling empirical evidence that the development and defence of understandings of citizenship education has never been, nor, as Joris (2025) suggests, should ever be the sole preserve of experts and theorists. Citizenship is inherently normative. Related debates, while fought more or less openly, are a defining feature of European societies at all levels and across types of government. These debates have involved a wide range of political and educational actors drawing not only on different sources of expertise (as theorised by Krick and Meriluoto, 2022), but also on political, emotional, and moral rationales. The papers thus invite us to take a close and critical look at instances of apparent consensus as inscribed in official legislation, regulations, and proclamations issued by states and international organisations. They also draw our attention to the potential precariousness of currently dominant conceptions and the work required to protect and/or reform them. While the papers in this special issue demonstrate the value of a historically informed approach in revealing the normativity, contestedness, and reformability of understandings of citizenship and of their relationship to education, we also hope that our endeavour will encourage further consideration of this relationship across the field of education.
